# Early Characterization of the Severity and Transmissibility of Pandemic Influenza Using Clinical Episode Data from Multiple Populations

**DOI:** 10.1371/journal.pcbi.1004392

**Published:** 2015-09-24

**Authors:** Pete Riley, Michal Ben-Nun, Jon A. Linker, Angelia A. Cost, Jose L. Sanchez, Dylan George, David P. Bacon, Steven Riley

**Affiliations:** 1 Predictive Science Inc., San Diego, California, United States of America; 2 Armed Forces Health Surveillance Center, Silver Spring, Maryland, United States of America; 3 Biomedical Advanced Research and Development Authority (BARDA), Assistant Secretary for Preparedness and Response (ASPR), Department of Health and Human Services (HHS), Washington, D.C., United States of America; 4 Leidos, McLean, Virginia, United States of America; 5 MRC Centre for Outbreak Analysis and Modelling, Imperial College London, United Kingdom; Pennsylvania State University, UNITED STATES

## Abstract

The potential rapid availability of large-scale clinical episode data during the next influenza pandemic suggests an opportunity for increasing the speed with which novel respiratory pathogens can be characterized. Key intervention decisions will be determined by both the transmissibility of the novel strain (measured by the basic reproductive number *R*_0_) and its individual-level severity. The 2009 pandemic illustrated that estimating individual-level severity, as described by the proportion *p*_*C*_ of infections that result in clinical cases, can remain uncertain for a prolonged period of time. Here, we use 50 distinct US military populations during 2009 as a retrospective cohort to test the hypothesis that real-time encounter data combined with disease dynamic models can be used to bridge this uncertainty gap. Effectively, we estimated the total number of infections in multiple early-affected communities using the model and divided that number by the known number of clinical cases. Joint estimates of severity and transmissibility clustered within a relatively small region of parameter space, with 40 of the 50 populations bounded by: *p*_*C*_, 0.0133–0.150 and *R*_0_, 1.09–2.16. These fits were obtained despite widely varying incidence profiles: some with spring waves, some with fall waves and some with both. To illustrate the benefit of specific pairing of rapidly available data and infectious disease models, we simulated a future moderate pandemic strain with *p*_*C*_ approximately ×10 that of 2009; the results demonstrating that even before the peak had passed in the first affected population, *R*_0_ and *p*_*C*_ could be well estimated. This study provides a clear reference in this two-dimensional space against which future novel respiratory pathogens can be rapidly assessed and compared with previous pandemics.

## Author Summary

The ever-increasing availability of timely, large-scale clinical episode data can, in principle, dramatically shorten the time required to characterize the transmission and severity of novel respiratory pathogens, which, in turn, can be used to inform key intervention decisions. We investigated 50 distinct military populations during the 2009 influenza pandemic to test the hypothesis that real-time encounter data combined with disease dynamic models can be used to jointly determine the transmissibility of the novel strain (described by the basic reproductive number *R*_0_) and its individual-level severity (described by the proportion *p*_*C*_ of infections that result in clinical cases). To illustrate the use of such a procedure, we simulated a future moderate pandemic strain with *p*_*C*_ approximately ×10 that of 2009, which demonstrated that even before the peak had passed in the first affected population, *R*_0_ and *p*_*C*_ could be well estimated. These results provide a clear reference in this two-dimensional space against which future novel respiratory pathogens can be rapidly compared, establishing a firm baseline for describing the relative severity of future emerging respiratory pathogens.

## Introduction

The increasing frequency with which large biomedical datasets are being made available is often referred to as the advent of “big data” [1]. There is substantial potential for the detection and characterization of emerging infectious diseases to benefit from the rapid availability of reliable big data [2], with one obvious opportunity being the reduction of our reliance on sentinel clinical surveillance systems for respiratory illnesses [3]. Given that sentinel systems are designed to estimate the frequency of clinical episodes, we should be able to improve our situational awareness during key phases of an outbreak by analysing detailed data on the clinical episodes themselves.

Novel strains of influenza emerge periodically [4–6] and pose substantial challenges to health planners in both civilian and military domains [7]. Primary among the issues that must be considered during the early stages of a potential pandemic are the appropriate strengths of possible interventions [8]. Effective interventions, such as vaccination, household-based quarantine and prophylactic use of antivirals, would likely eliminate a substantial proportion of onwards transmission from any single infectious individual [9]. However, these interventions incur considerable costs [10, 11], which may not be justified.

**Fig 1:**
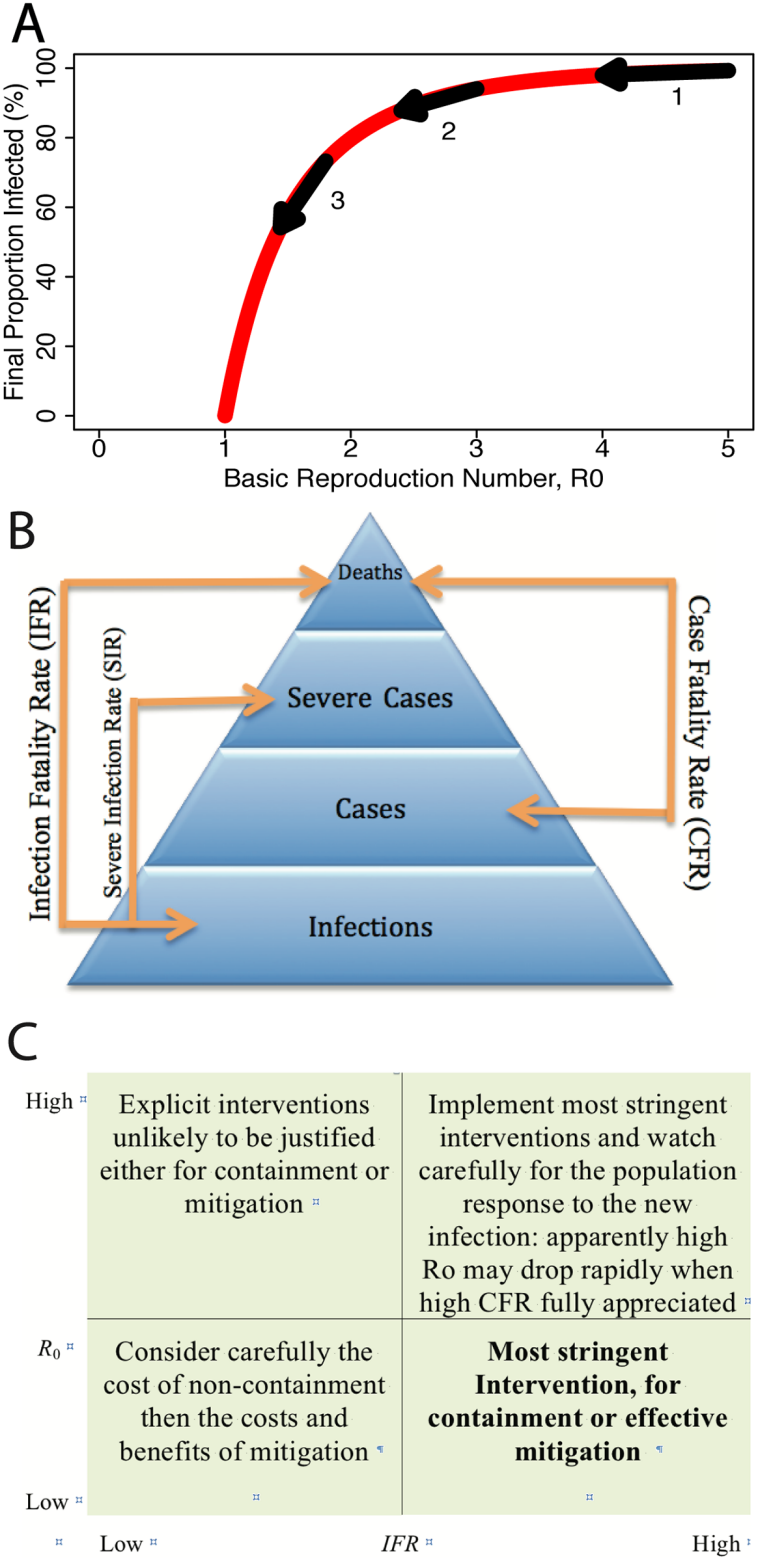
Transmissibility and severity of pandemic influenza. **a** Relationship between the total number of individuals infected and the basic reproductive number *R*_0_. Arrows show the non-linear effect of a 20% reduction in transmission: at lower reproductive numbers, the same intervention is much more effective. **b** Severity pyramid for infectious disease. The strength of symptoms and ability to detect cases increases with each level in the pyramid. **c** Conceptual two-dimensional classification of pandemics in terms of basic reproductive number (*R*_0_) and severity (*p*_*C*_), illustrating the likely impact of interventions, depending on where the outbreak falls in this parameter space (see main text).

The transmissibility of an emergent strain in a particular population is quantified by the basic reproductive number *R*_0_, defined to be the average number of secondary cases generated by one typically infectious individual in an otherwise susceptible population [12]. If interventions are in place before the arrival of a new virus, as they are likely to be for many populations during a moderate or severe pandemic, their transmission-blocking efficacy can be thought of as proportional reduction in *R*_0_. The same proportionate decrease in *R*_0_ is much more effective in reducing the overall cumulative attack rate (CAR) for lower absolute values of *R*_0_ than for higher absolute values (Fig 1A). Thus, estimates of *R*_0_ for pandemic influenza in the range 1.5 to 3 [13, 14] are important because they imply a high population efficacy for interventions that reduce *R*_0_ by only modest proportions [9, 15], even if containment [16, 17] is not achieved.

Although reductions in the transmissibility of an infectious disease are almost always desirable, the 2009 pandemic demonstrated clearly that the degree to which costly interventions are justified is also highly dependent on the individual-level severity of the emergent strain. Despite being more difficult to measure than the case-based statistics, the clearest and most transferrable measures of individual-level severity are those that use infection as their denominator. For example, the infection fatality rate (IFR) is defined to be the risk of death conditional on infection (Fig 1B) [18]. If the IFR is low, as was the case during the 2009 pandemic [19], expensive interventions are unlikely to be justified. However, IFRs vary considerably by strain: in terms of IFR, both the 1918 H1N1 [20] and avian H5N1 (in humans) [21] are likely orders of magnitude more severe than the 2009 pandemic strain. Since 2009, one-dimensional severity scales for influenza pandemics based on the severity [22] have been replaced by more nuanced two-dimensional approaches which are able to reflect the importance of both individual-level severity and transmissibility [23, 24] (Fig 1C).

Here, we address the principal epidemiological challenge implied by these revised guidelines for pandemic response: how best to characterize the transmissibility and individual-level severity of an emergent strain in the shortest possible time. We extended a previous study of influenza-like-illness (ILI) in the US military population [25] by developing a parsimonious epidemic model of both infection and clinic attendance in multiple similar populations of approximately known sizes. Our objective was to be able to improve the speed with which key disease dynamic properties could be estimated from high quality clinical episode data, by extracting the maximum possible information from early affected populations. Essentially, the shape of the epidemic curve in each population allows us to fit a model and to infer unobserved numbers of infections.

## Methods

### Data

We extended our previous analysis of the Defense Medical Surveillance System (DMSS) data to characterize the relationship between ILI cases and severe influenza at the level of an individual military population, across the duration of the 2009 pandemic. We extracted 21,573 clinical influenza episodes between April 1 2009 and June 30 2010 (using the most specific available definition of ILI [25, 26]). Within these episodes, 315 cases were coded as severe influenza (ICD-9 code 487). Each episode was assigned to a military population by the zip code (MPZs) based on the clinic in which the episode occurred. In our analysis, we focus on the top-50 installations in terms of total number of ILI cases. These captured 13,794 episodes of clinical influenza (64%) and 254 cases of severe influenza (81%).

We used the 5-digit zip code of the reporting clinic as a proxy with which to define military installations: we do not explicitly represent military installations or bases, rather, we assume that case reports from the same zip code are, effectively, from the same population.

### Models

We considered a set of independent deterministic transmission models, one for each military installation with a constant background rate of clinical report. For each, we solved the following set of equations:

$$\frac{dS}{dt}=-\frac{\beta (t)SI}{N_{total}},$$ (1)

$$\frac{dI}{dt}=\frac{\beta (t)SI}{N_{total}}-\frac{I}{T_{g}},$$ (2)

$$\frac{dR}{dt}=\frac{I}{T_{g}},$$ (3)

$$\beta \left(t\right)=\{\begin{array}{cc} \beta_{A} & \text{if}\;t_{1}\leq t<t_{1}+\Delta t\\ \beta_{B} & \text{otherwise} \end{array}$$ (4)

where *S* represents the number of susceptible individuals, *I* is the number of infectious individuals, *R* is the number of recovered individuals, *N*_*total*_ = *S* + *I* + *R* is the total active duty population size at each installation, and *T*_*g*_ is the generation time, or in this model, the average time of infection, which we keep fixed at 2.6 days. We found that estimates of *R*_0_ and *p*_*C*_ were relatively insensitive to the value of *T*_*g*_ assumed (See Supplementary Materials, S3 Fig).

The incidence (*I*_*R*_) is given by $-\frac{ds}{dt}$, which computationally, is estimated by:

$$I_{R}=p_{C}\;\int_{t_{s}}^{t_{f}}\;\frac{\beta (t)S(t)I(t)}{N_{total}}dt$$ (5)

where *p*_*C*_ is the proportion of the infectious active duty population that present themselves to a clinic with ILI-small symptoms, and the integral runs over a week from *t*_*s*_ to *t*_*f*_.

The time-dependent term, *β*(*t*), changes from *β*_*A*_ to *β*_*B*_ at time *t*_1_ and returns to *β*_*A*_ after an interval Δ*t*. Since *β* = *R*_0_/*T*_*g*_, and the generation time, *T*_*g*_ is fixed, this is equivalent to allowing the basic reproduction number, *R*_*A*_, to change at some point in time, *t*_1_, to a new value *R*_*B*_. Intuitively, this definition makes sense if we imagine some mechanism, such as school closures on installations, the deployment of troops, or some other behavior modification to drive the effective contact rate down, and, hence, *R*_0_. For purposes of generality, however, we did not impose any requirement that *R*_0_ decrease at this time.

Even during a pandemic, there are reasons other than influenza infection for cases to present as ILI. Therefore, we also included a noise term. It was implemented as a constant added to the model output for incidence during the optimization procedure, resulting in a total of seven parameters (*β*_*A*_, *β*_*B*_, *t*_1_, Δ*t*, *p*_*C*_, a background ILI noise term, and the week of ILI pandemic onset).

We determined the joint posterior distribution for the model parameters using a Metropolis-Hastings Markov Chain Monte Carlo (MCMC) procedure [27]. For each base we simulated four MCMC chains each with 10^8^ steps and a burn time of 2.5 × 10^7^ steps. At each step a new set of parameter values was sampled from a log-uniform distribution (the minimum and maximum allowed values for the parameters are summarized in S1 Table). Using this set of candidate parameters we generated a profile for the base and calculated the log-likelihood of the profile. The values of the new and previous log-likelihood was used in a standard rejection method to determine if the move should be accepted or rejected. Our MCMC chains had a typical acceptance rate of 20–40% and an effective sample size that was in the 200–2000 range (depending on the base profile and the parameter).

We note that with this model structure, we make no strong assumptions about the variation in infectiousness of individuals, other than that the distribution of infectiousness is approximately constant and well described by its mean. For example, it can be shown mathematically that the presence of an unobserved additional infectious class, always present in a fixed proportion to the observed infectious class, would not affect our parameter estimates or model projections.

## Results

The relationship between mild and moderate clinical cases and severe clinical cases can be measured directly from episode data (Fig 1). We used the ratio *p*_*S*_ of severe influenza cases to ILI cases as a proxy for the relationship between layers of the severity pyramid from clinical cases upwards. The ratio *p*_*S*_ could be described directly from the episode data, with our analyses suggesting that, although the average of *p*_*S*_ varied little through time, there was substantial variation by military population (defined by zip code, MPZ, see MATERIALS AND METHODS and S1 Fig). For the period of the study, there were 315 severe influenza cases out of a total of 21,573 ILI cases, giving an average of 1.46% (95% CI ±0.16). Although there was some evidence from fitting a smooth regression term that this ratio varied during the period of the epidemic, the maximum amplitude of variation was small and an odds ratio of one fell within the 95% confidence interval for most of the year. However, non-overlapping binomial confidence bounds for point estimates of *p*_*S*_ for individual MPZs suggested that differences between populations were significant and could not be explained simply by chance.

**Fig 2:**
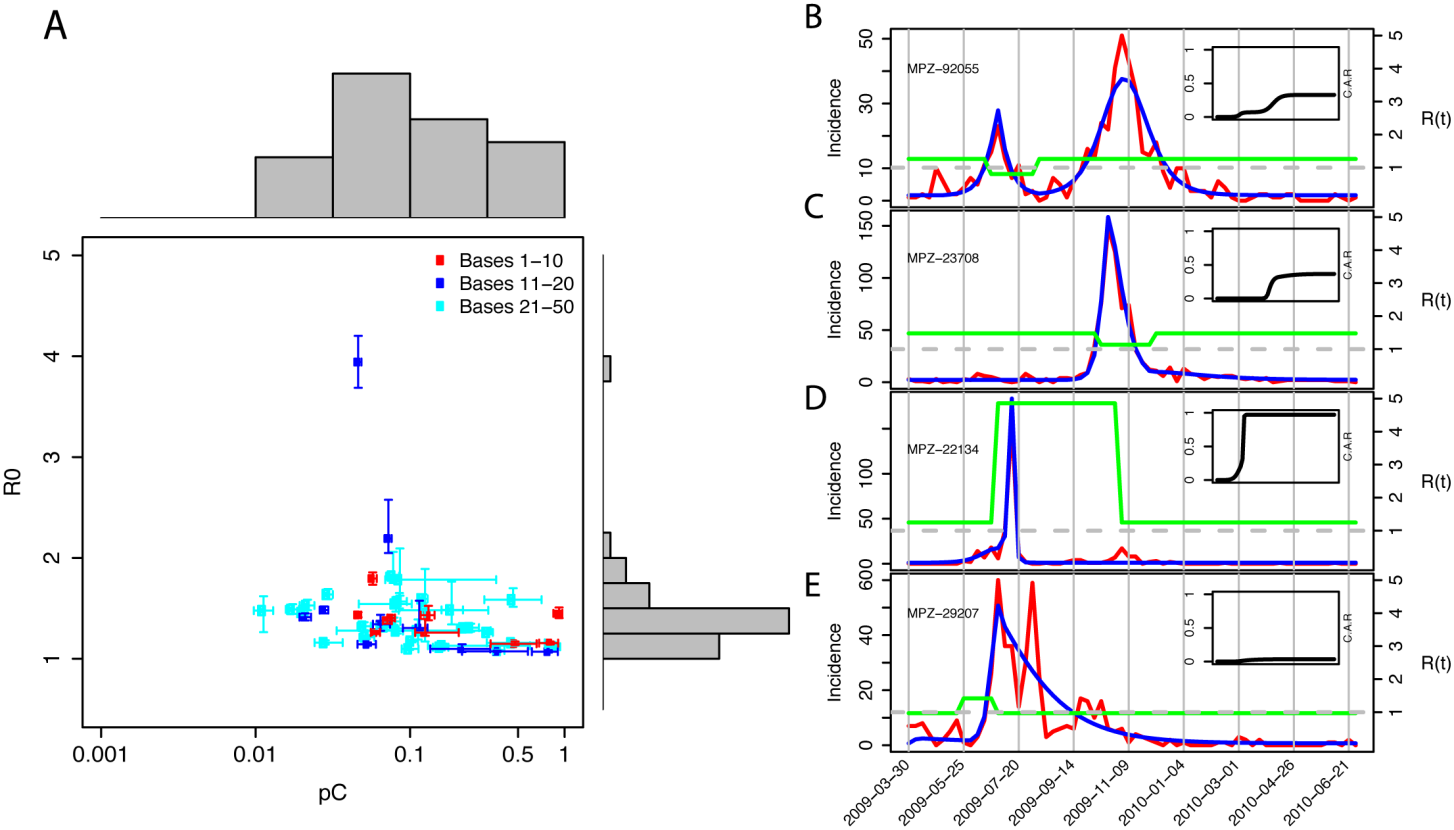
Characterization of overall severity and individual base fits. **a** Estimates of *R*_0_ versus *p*_*C*_ for top-50 military installations. The ten installations with the largest number of ILI cases are colored red, installations 11 through 20 are colored blue, and the remaining 30 bases are colored cyan. The grey box denotes the 40 installations with the smallest area in *p*_*C*_-*R*_0_ space. The histograms along the top and right show the distribution of *p*_*C*_ and *R*_0_ values, respectively. **b-e** Incidence rates for four military installations (red line), with model fits overlaid (blue line), illustrating: **b** a two-peak profile; **c** a single-peak profile; **d** an anomalously high and narrow profile; and **e** a complex profile. The green line shows the value of the basic reproduction number and the horizontal dashed grey line marks the critical value of 1.0. The inset in each panel shows the cumulative attack rate for the same time period.

To describe the key features of the lower portion of the severity pyramid, we extended a previous mechanistic model of influenza transmission in these MPZs [25]. In our earlier work, we assumed a value of *p*_*C*_, the proportion of infections that resulted in ILI, and fitted only *R*_0_. Here, we estimate *p*_*C*_ and *R*_0_ jointly, by using known approximate size for each population (see MATERIALS AND METHODS, Fig 2, S2 Table). As expected, estimates for the basic reproduction number *R*_0_ were similar to those in our previous work [25], although there were some exceptions. Seven of the ten largest MPZs formed a distinct cluster within the *R*_0_-*p*_*C*_ space, within the ranges: *R*_0_ between 1.12–1.53 and *p*_*C*_ between 0.052–0.15. Visually, the fit of these models to ILI incidence data was good (S2 Fig).

The mechanistic model was able to capture a variety of epidemic profiles. For example, among the seven bases with a large number of ILI cases closely clustered in *p*_*C*_−*R*_0_ space, the degree to which each exhibited a double-peak epidemic profile varied considerably. MPZ 92055 (Camp Pendleton, CA; Fig 2B) exhibited a clear two-peak profile with a substantial early wave during the spring of 2009 followed by a similar-sized fall wave. Our MCMC parameter estimation routine (see MATERIALS AND METHODS) found solutions (S2 Table) in which the first peak occurred because of a drop in *R*_0_ from supercritical 1.14 (95% CI 1.11–1.16) to pracitcally zero (0.021 median and 95% CI 0.016–0.086). The second peak occurred once *R*_0_ had returned to its original level and the pool of susceptible individuals was depleted; the shape of the second peak for Camp Pendleton was determined solely by the characteristics of the remaining susceptible pool as saturation occurred. Conversely, reports of ILI from MPZ 23708 (Portsmouth, VA; Fig 2C) clustered into a single clearly defined epidemic profile, with a peak that appeared sharper than that of Camp Pendleton. The optimal solution for Portsmouth included a drop in *R*_0_ from 1.40 (95% CI 1.35–1.48) to 1.12 (95% CI 1.08–1.17) that coincided with the depletion of susceptibles. This drop in transmissibility was reversed shortly afterwards to permit a slightly larger right tail to the incidence pattern.

Although it was reassuring that—despite substantial differences in their incidence profiles—estimates of both *R*_0_ and *p*_*C*_ clustered tightly for many MPZs, this was not always the case. We consider here four populations out of the ten that fell outside the central area of *p*_*C*_—*R*_0_ space denoted by the grey rectangle in Fig 2A. Our estimate of *R*_0_ of 2.65 (95% CI 2.50–2.78) for MPZ 22134 (Quantico, VA; Fig 2C) was the highest of all 50 military installations, and resulted in a much higher cumulative attack rate (CAR, see inset in Fig 2D). Given that estimated values of *p*_*C*_ of 0.025 (95% CI 0.022–0.027) for Quantico were within the range seen for other large bases, it is plausible that, because of the large size and training focus of this particular population, this model fit reflected genuine differences in epidemic dynamics: the pathogen spread more rapidly here because of the population structure. However, the same is probably not the case for MPZ 29207 (Fort Jackson, SC; Fig 2E). The best fit model parameters for Fort Jackson produced an unusual epidemic profile with a very rapid rise followed by a slow exponential decay; only reproducing observed ILI patterns with a very high value for *p*_*C*_ 0.88 (95% CI 0.41–0.99). Hence, the estimated CAR for Fort Jackson was much lower than those estimated for most other MPZs, and probably not realistic. Population MPZ 28130 (Fort Bragg, NC) had similar dynamics to those of Fort Jackson, in having a high *p*_*C*_ (0.87) and low *R*_0_ (1.07), also likely driven by an aborted epidemic. Finally, for MPZ 39534 (Keesler Air Force Base, MS), a large training facility, our model again finds a high value of *p*_*C*_ (0.57) and low value of *R*_0_ (1.09). Overall, eight of the ten outliers in Fig 2A are characterized with medium-to-large *p*_*C*_ values and low-to-average *R*_0_ values with the remaining two outliers having large *R*_0_ values and low *p*_*C*_ values.

**Fig 3:**
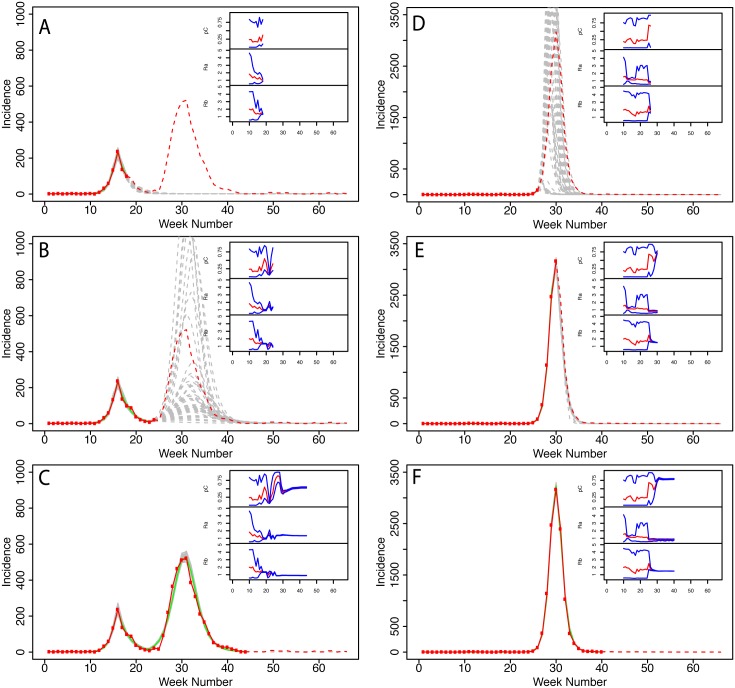
Characterizing a model strain of pandemic influenza in real time for a single population. **a-c** Three frames illustrating stochastic predictions for an outbreak based on parameters that give a typical two-peak profile (such as that of MPZ-92055), but increasing *p*_*C*_ by about an order of magnitude. The grey lines give individual simulation realizations while the green line shows the ensemble average. The data contributing to the realizations is marked in red. The insets show the evolution of computed *p*_*C*_, *R*_*A*_, and *R*_*B*_ as more simulated data are added to the predictions. The red line is our median estimate and the blue lines are the 95% confidence intervals. **d-f** Analogous frames for a typical single-peaked outbreak such as that of MPZ-23708, but again increasing *p*_*c*_ by about an order of magnitude.

To assess the likely utility of this type of data stream to improve the speed of characterization of a new strain in real time, we simulated infections and clinical attendance in two illustrative populations and then estimated key parameters at different time points (Fig 3). Parameter values for the simulated incidence were chosen to simulate a double and single peak profiles similar to the 2009 ILI profiles of MPZs 92055 (Camp Pendleton, Fig 3A–3C) and 23708 (Portsmouth VA, Fig 3D–3F), other than that we used a of *p*_*C*_ about ten times greater, to increase the overall incidence. We considered first the simpler single peak profile (Fig 3D–3F and S2 Movie in the Supplementary Materials). Initially, during the exponential phase of the epidemic, unbiased point estimates of *R*_0_ were possible but only with considerable uncertainty (Fig 3D). However, with this limited data, it was not yet possible to estimate *p*_*C*_ or to make predictions for the peak number of ILI cases. Once the growth in incidence had slowed and was clearly sub-exponential, uncertainty in parameter estimates and model predictions was greatly reduced (Fig 3E). In relative terms, little additional information was contained in the additional data gathered between the second and the third time points (Fig 3F).

We also considered a more challenging two-peak epidemic profile (Fig 3A–3C and S1 Movie in the Supplementary Materials). The model initially captures the profile of the first wave well, but has no knowledge that a second, larger peak will occur (Fig 3A). However, when the second wave begins the model is capable of immediately revising its favored trajectory and estimating the duration and timing of the second peak. As soon as the exponential phase of the second peak begins to saturate, the model is also able to predict the amplitude of the second peak and the overall size of the epidemic. The uncertainty in the model predictions as a function of time during the outbreak is considered more thoroughly in the Supplementary Materials (S4 Fig).

## Discussion

Sentinel surveillance systems [28] that are designed to estimate the number of clinical episodes currently occurring as a result of respiratory infection will soon become obsolete in many healthcare systems. Near complete data on those episodes are already being gathered electronically in real-time at the point of care and will soon be made available for analysis within a short period of time. Therefore, given the rapid global spread of 2009 pH1N1, it is likely that there will be an opportunity to characterize the next pandemic strain using mathematical models and “big data” from clinical episode reporting systems in highly-connected, well-resourced populations such as the US military.

The proportion *p*_*C*_ of infections that become clinical cases is a key unknown for any population for which robust clinical episode data are available and can be estimated using a parsimonious mechanistic model. Although an advantage of large episode datasets over data from disjoint sentinel systems is that the same process captures both mild and severe illness, episode datasets contain no information on mild or entirely asymptomatic infections. Therefore, we used knowledge of the transmission process, captured by a mechanistic model, to estimate the number of infections and hence calculate *p*_*C*_. Our specific methods will need modifying for less homogeneous populations and for epidemics for which pre-existing immunity is an issue. However, the general approach of using parsimonious mechanistic models to augment timely clinical episode data by describing the underlying disease dynamics is likely to be useful in many settings.

There was a substantial degree of variation in our estimates of *p*_*C*_, which could arise from the process of reporting these cases, or from structural uncertainty in the model, and may cast doubt on the utility of our results. However, given that good health information has been available for our study population for many years, we would argue that it is likely to remain available for many years and that our results establish a clear baseline that will be of considerable use during the next pandemic. Should a similar future study of an emerging influenza strain find significantly higher values for *p*_*C*_, even against this background of unexplained population-level variance, that would be very strong evidence that the new strain was significantly more severe than the 2009 strain. Model variants could be tested on both old and new datasets to reduce the potential impact of structural bias on the overall conclusion.

More generally, variation in our results raises the challenging concept that key disease parameters such as *R*_0_ and *p*_*C*_ are not universal for respiratory infections and, as with many other ecological descriptors, depend on the time, host, and environment. Although this must be true to some degree, the public health significance of measurable variation in parameters such as *R*_0_ is less clear [29]. This study and other recent work [30] is starting to tease out good explanations for location-to-location variation for influenza. The concept of location-specific transmissibility is much more widely accepted for other infections such as HIV [31].

The inverse of *p*_*C*_ is the multiplier required to calculate the number of infections when the number of cases are known. Our estimate of the multiplier is between x7 and x19 (based on median point estimates for *p*_*C*_ for the most similar seven populations in the ten largest ILI profiles). These values are substantially lower and have less associated uncertainty than estimated multipliers for the civilian population in the US [32] and elsewhere [33]. This apparent inconsistency is likely driven by the slightly different objectives of our study compared to other examples. We were not attempting to directly link laboratory-confirmed cases with the total number of clinical cases: it is notoriously difficult to obtain temporally unbiased laboratory data for an epidemic because the capacity for testing is often highly constrained. Rather, we have described the relationship between all clinical cases (according to a consistent definition and a near-complete dataset) and model-estimated infections. Therefore, for populations for which clinical cases can be observed directly, the infections-to-cases multiplier we have described here is likely more appropriate.

Good knowledge of *p*_*C*_ (or the multiplier) early in a pandemic would remove key uncertainties and allow the IFR to be estimated directly from case data. Together with knowledge of transmissibility, an accurate estimate of the IFR would allow the formulation of an appropriate response [23, 24]. In particular, if transmissibility is low, there would be a reasonable chance of future local containment, or highly effective mitigation [9], and knowledge of the IFR would become crucial. The benefits arising from either containment or effective mitigation for a high IFR could be enormous and thus justify the rapid allocation of substantial resources.

We chose to present individual-level severity in terms of the IFR, rather than the case fatality rate (CFR). For any given population and reporting system, the IFR is closely related to the CFR. However, for most respiratory pathogens many infections do not become cases ensuring that the CFR is substantially lower than the IFR. Also, the probability of becoming a recorded case, conditional on a specific set of symptoms, varies tremendously for civilian populations from place to place within the same country and also from country to country [18].

Generally, peaks in incidence occurred because of the partial depletion of the susceptible population. However, our model results provide hints where this may not have been the case. For 10 of the top-50 military installations (i.e., 20%), the peak in incidence coincided with a drop in *R*(*t*). In six of these cases, the drop was modest, only just decreasing to below 1.0; however, for four installations (92055 (first wave), 39534, 87117, and 96319), the drop was significant. Without knowledge of the personnel activities during the 2009–2010 interval, we could only speculate on the possible behavioral changes that might have been responsible for these variations. We can, however, rule out a change in the total population at each base; a reduction of which could drive the extinction of the outbreak. To do this, we estimated the total number of visits to each clinic from the DMSS database, regardless of diagnosis, and used it as a proxy, proportional to the total population of that installation. Although we identified several installations where this number varied significantly during 2009–2010, none of them coincided with the four cases for which *R*(*t*) dropped substantially at the peak. Thus, we can rule out variations in base population as a driving factor behind the outbreak dynamics.

The model was able to reproduce the two wave patterns (spring and autumn) seen at a number of the installations (See Supplementary Materials, S2 Fig). Generally, this was accomplished by modulation of *R*(*t*); At the peak of the first wave, *R*(*t*) decreased, rising again just prior to the start of the second wave (e.g., MPZ-98431). In one case (MPZ-92134), *R*(*t*) increased during the second wave to accommodate a second wave that was larger than the first.

Our results suggest a substantial degree of variation in the proportion of each base infected and in per infection severity. If these differences are maintained from one influenza season to the next, knowledge of that variation could be valuable for fine tuning the allocation of scarce resources such as anti-virals and pre-pandemic vaccine. However, there were a number of substantial sources of uncertainty that we were not able to represent to the best possible accuracy in these analyses (see below). Therefore, we intend to focus more on the characterization of intrinsic transmissibility differences per population in future studies when data are available for multiple seasons.

Perhaps the most significant source of uncertainty in our results lies in the estimate for the total population at each military installation (*N*_*total*_), the “denominator data”. Our method for estimating these sizes relied on the use of the total number of visits to a clinic for all causes as a proxy for the total number of active duty personnel at that location [25]. Although the linear relationship between this and the publicly-released population sizes of the installations was clear, there were notable exceptions. While *N*_*total*_ may not be well known for all installations within the civilian domain, it is, or can be well determined within the military, and, thus, can be accurately estimated when necessary.

Additionally, we did not include age-classes explicitly in this study, largely because of a lack of good denominator data. We examined age-specific incidence for each population and found no material differences (not shown). Hence, there was no need to test an age-stratified version of the model for this population. However, it is likely that the epidemic dynamics we observe in our study population were influenced by age effects in the surrounding civilian population. Therefore, we suggest that our absolute estimates of transmissibility likely reflect the wider population while our results for severity are specific to the age group within our study. While this age group is not traditionally the one most affected by influenza, it is an economically important age group. Also, our study population can form a valuable bench mark for year-on-year or pandemic-on-pandemic comparative assessment of severity. While age-dependent effects are likely to be less important within the military population than in civilian populations, due to a narrow range of ages in the military (18–45 years old), they clearly will have some impact. Again, as with the “denominator data,” age-specific information for each installation is undoubtedly available to military planners and could be incorporated into our analysis.

The visualization of model-derived evidence is an important aspect of the communication of key public health messages. Our visual descriptions of the simulation study presented the following items in a fully integrated format: currently available data, model fit, key model parameter estimates, and model projections. We suggest that this approach to the communication of real-time analysis during an outbreak may facilitate the comparison of results from parallel model-based studies. Although we have used only a single flexible model in this study, there is no reason that this visualization approach could not be extended to model ensembles [34].

It could be argued that public health intelligence based on a proprietary military data source is of only limited utility. However, in an era where the value of big data is recognized, we must accept that the highest quality and most timely data will very rarely be immediately open access. Therefore, it is important not to fully conflate the need for increased access to timely data and the need to extract the maximum actionable information from such data. An accurate assessment of a novel influenza strain would be of considerable value independently of the detailed data on which the assessment is made. We believe that the analysis and data presented here—together with the structure of the author group—suggest a genuine commitment to making better use of high-value national resources for improved health decision making across both civilian and military populations.

## Supporting Information

S1 Table Minimum and maximum values for the seven parameters used in the study. (PNG)

S2 Table Model fit parameters for the top-50 MPZs. (PDF)

S1 Fig Relationship between severe influenza and influenza-like-illness (ILI). **a** shows: ratio of severe influenza cases to ILI (*p*_*S*_) per week (red line, left y-axis); *p*_*S*_ for the 20 largest military populations by zip code (MPZ) with the y-location of each MPZ defined by the peak ILI incidence (vertical lines show 95% binomial confidence bounds); and total number of ILI per week (grey lines, renormalized to a maximum value of six for convenience of comparison). **b** shows a fitted spline from a logistic generalized additive model of date as risk factor for an ILI being severe influenza (shaded region is 95% confidence interval).
(EPS)

S2 Fig Influenza incidence (i.e, the number of reported ILI cases per week) observed (red) and model fit (blue) as a function of time during the 2009 pandemic for the top-50 military installations. The value of the basic reproduction number is shown in green. A value of 1.0 is indicated by the dashed grey line. The inset shows a box plot of *p*_*C*_ and *R*_0_, obtained from the MCMC chain, with the whiskers extending to the extreme values. The military installations are ordered by the total number of ILI cases reported.
(PDF)

S3 Fig The sensitivity of the model results to the particular value of *T*_*g*_ chosen was explored by computing solutions with *T*_*g*_ assumed to be 20% higher (3.12 days, green) and 20% lower (2.08 days, blue) than the canonical value of 2.6 days (red). Panel A summarizes the value of *R*_0_ obtained for each military installation (represented as base index for simplicity—see S2 Table to transform from base index to MPZ) for the three values of *T*_*g*_, demonstrating that our results are, for the most part, not sensitive to the precise value of *T*_*g*_ assumed. One notable exception is base 23604 (base index 30), which corresponds to Ft. Eustis, an army school located in Newport News, Virginia. Panel B summarizes the values of *p*_*C*_, also estimated using the three values of *T*_*g*_, and again demonstrating relatively little sensitivity. For both *R*_0_ and *T*_*g*_, the trends from one base to the next generally track well. Additionally, and as intuitively expected, increasing *T*_*g*_ correlates with an increase in *R*_0_ and a decrease in *p*_*C*_. Finally, in panel C, we compare the median *AIC*_*c*_ scores computed for each model solution, which suggests that the quality of the model is not obviously affected by our choice of *T*_*g*_.
(EPS)

S4 Fig The uncertainty of the model predictions is shown in two ways. (a) The 95% confidence intervals are shown for military installation 23708 as a function of time by running the fitting procedure using data from the first three weeks, then four weeks, then five weeks, etc., until the full 66 weeks are used. The red curve shows the mean value and the grey line, together with the y-axis on the right-hand-side indicates the ILI profile. Thus, the accuracy substantially improves one week after the exponential rise portion of the outbreak is observed. (b) A second measure of the uncertainty can be estimated from the model’s ability to predict the peak week within ±1 week (e.g., [34]). Here we show results using 250 random selections from the MCMC chains for the same MPZ (23708). The panels show histograms for the MCMC forecast peak timing for predictions made with 3, 6, 9, etc., data points. The actual peak for this installation occurred at week 43 and is marked in green. The red vertical line marks the average of the MCMC ensembles (each of which is shown in blue). Thus, we conclude that only about 3 weeks before the peak occurs do all the predictions collapse down to what will be the observed peak week. This is consistent and complementary to the results shown in (a).
(PDF)

S1 Movie Movie (from which the frames D-F in Fig 3 were extracted) illustrating stochastic predictions for an outbreak based on parameters that give a typical two-peak profile (such as that of MPZ-92055), but increasing *p*_*C*_ by about an order of magnitude. The grey lines give individual simulation realizations while the green line shows the ensemble average. The data contributing to the realizations is marked in red. The insets show the evolution of computed *p*_*C*_, *R*_*A*_, and *R*_*B*_ as more simulated data are added to the predictions. The red line is our median estimate and the blue lines are the 95% confidence intervals.
(MOV)

S2 Movie Analogous to S1 Movie but for a typical single-peaked outbreak such as that of MPZ-23708, and again increasing *p*_*c*_ by about an order of magnitude. Frames A-C in Fig 3 were extracted from this movie.
(MOV)
