# Supplementary figures and images for: Early Characterization of the Severity and Transmissibility of Pandemic Influenza Using Clinical Episode Data from Multiple Populations
Source: PLoS Comput Biol. 2015 Sep 24;11(9):e1004392. doi: 10.1371/journal.pcbi.1004392 (PMC4581836; doi:10.1371/journal.pcbi.1004392)

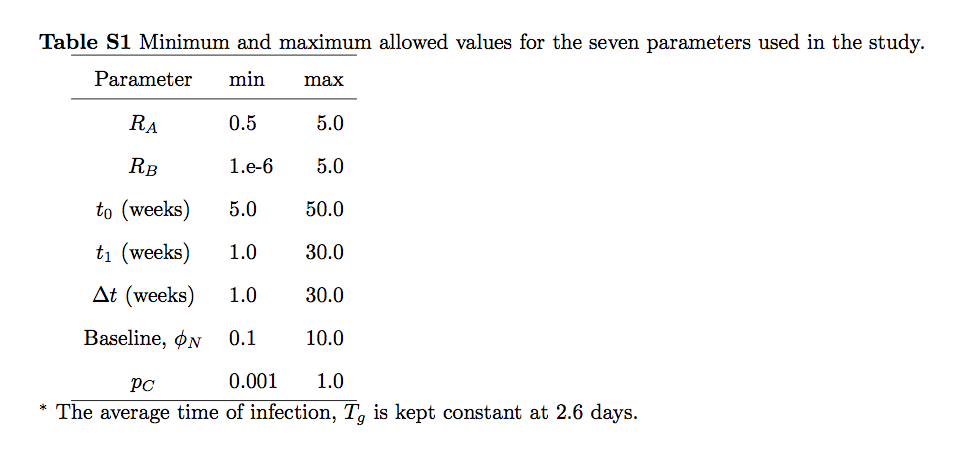

Supplement: S1 Table — (PNG) [file pcbi.1004392.s001.png]

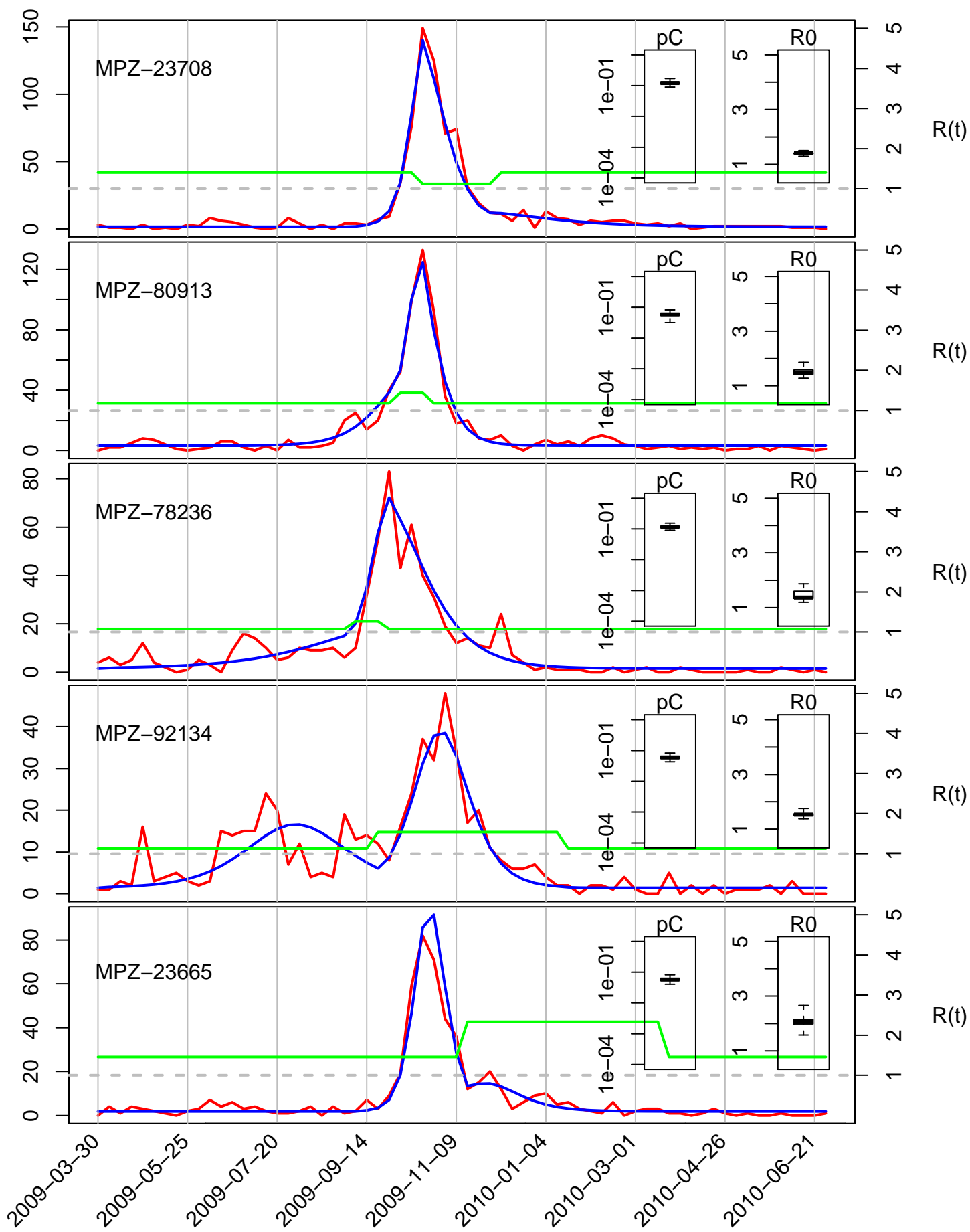

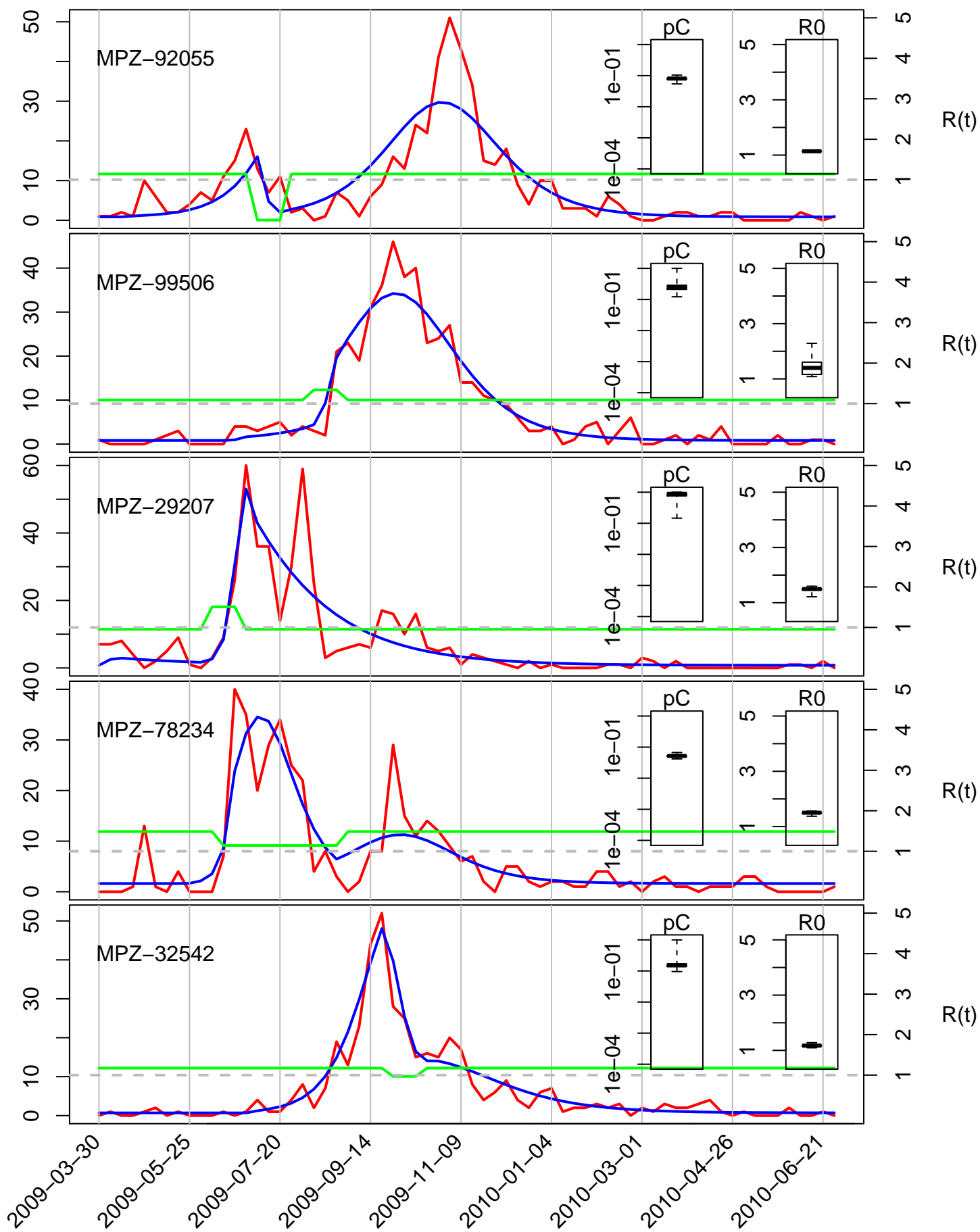

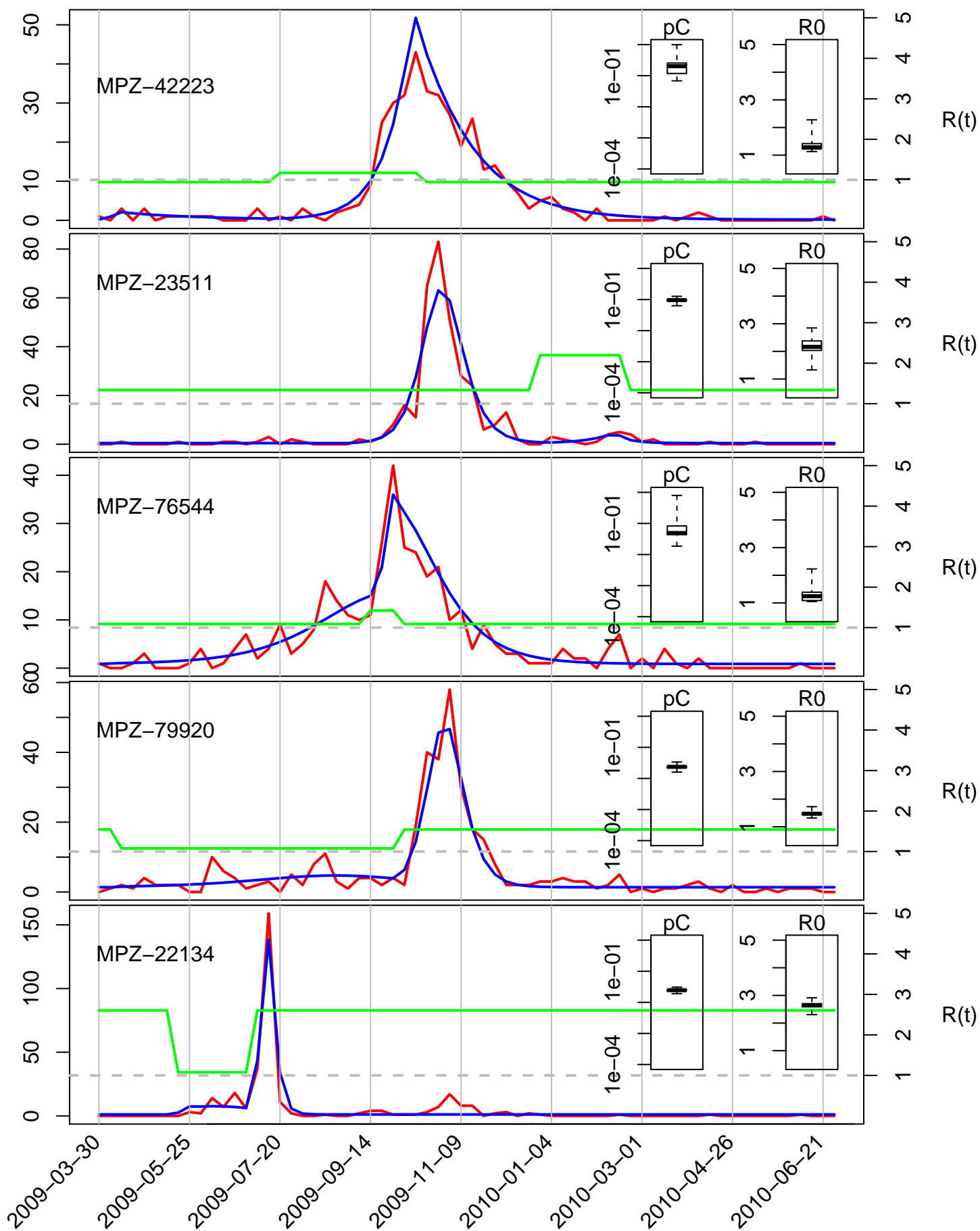

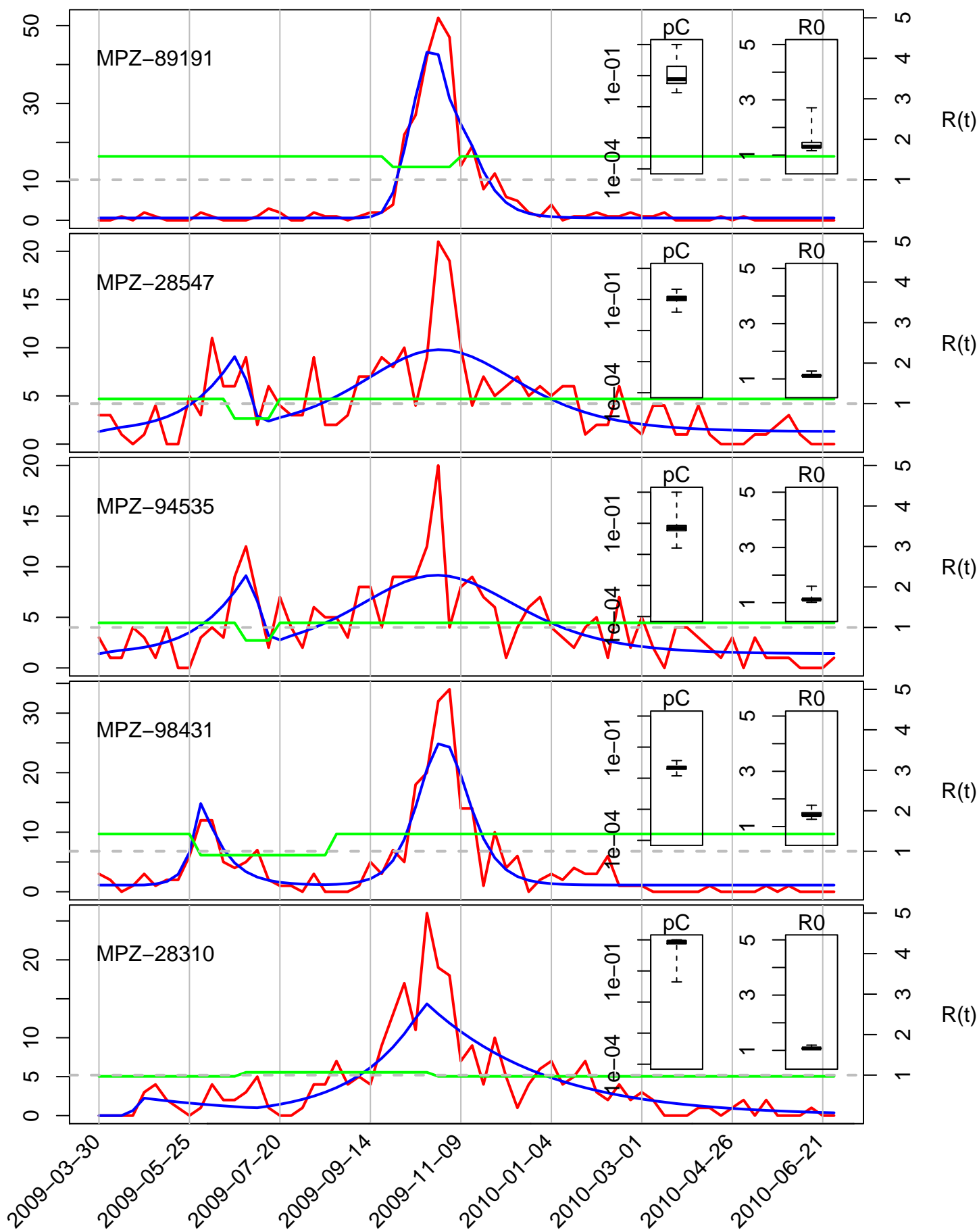

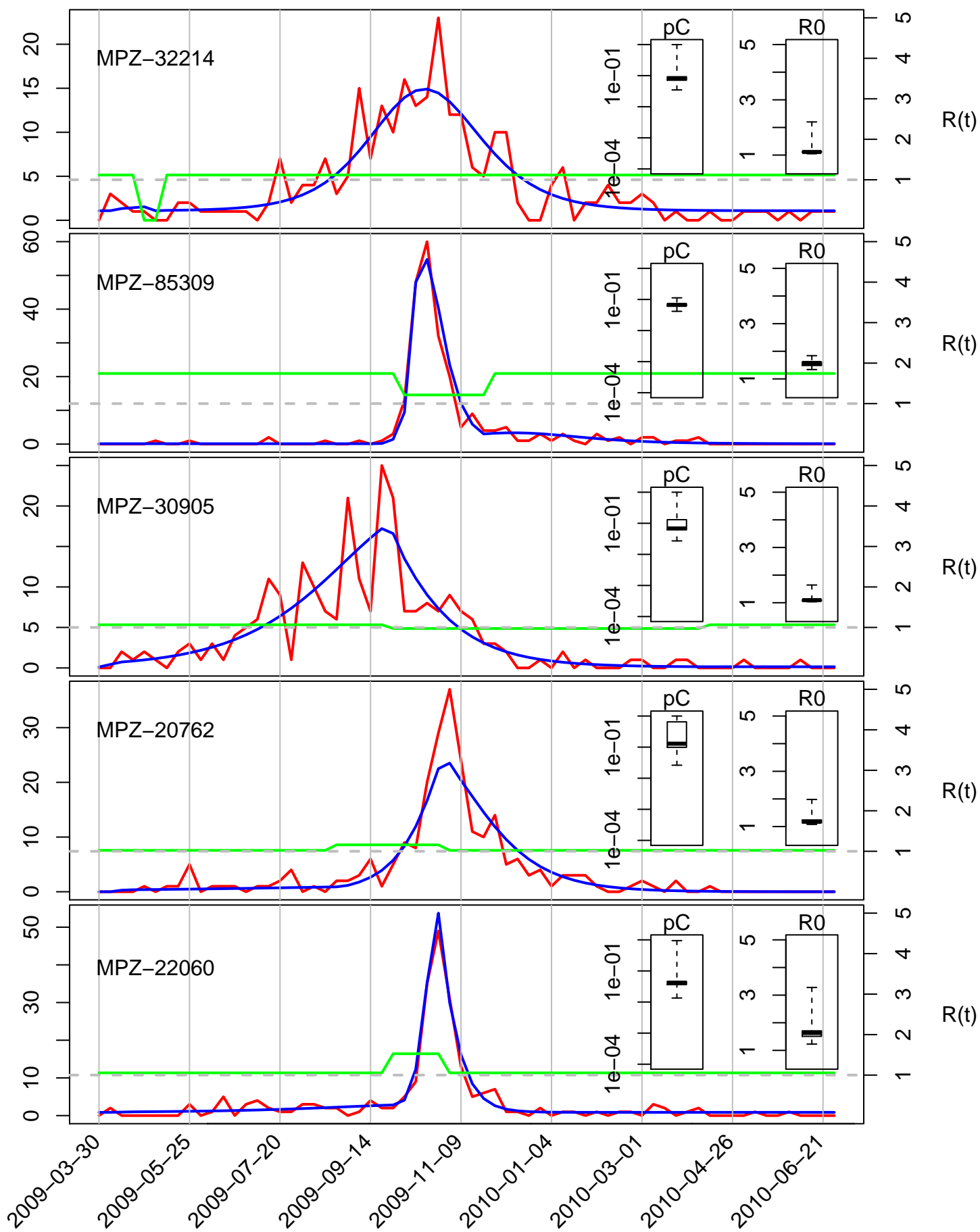

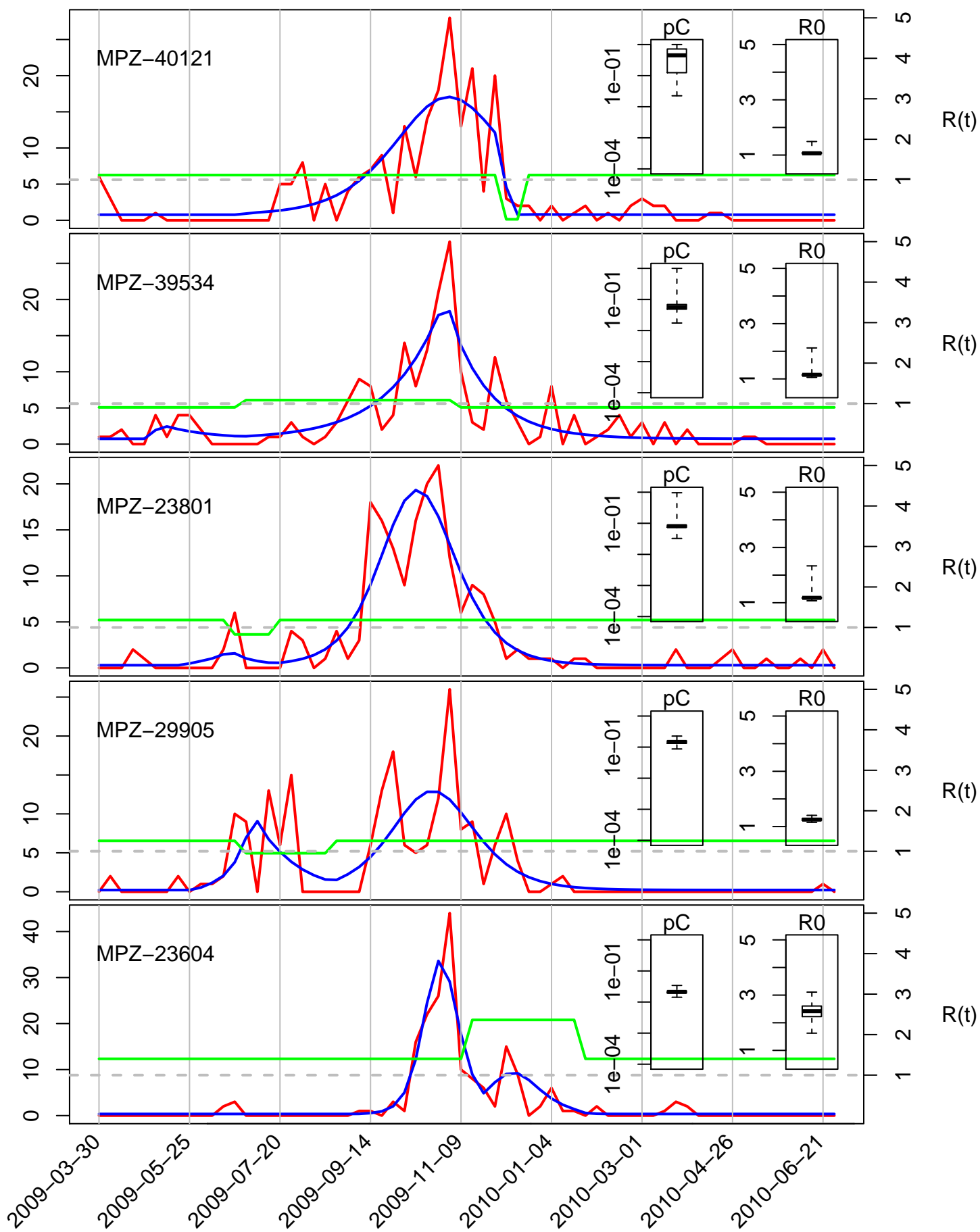

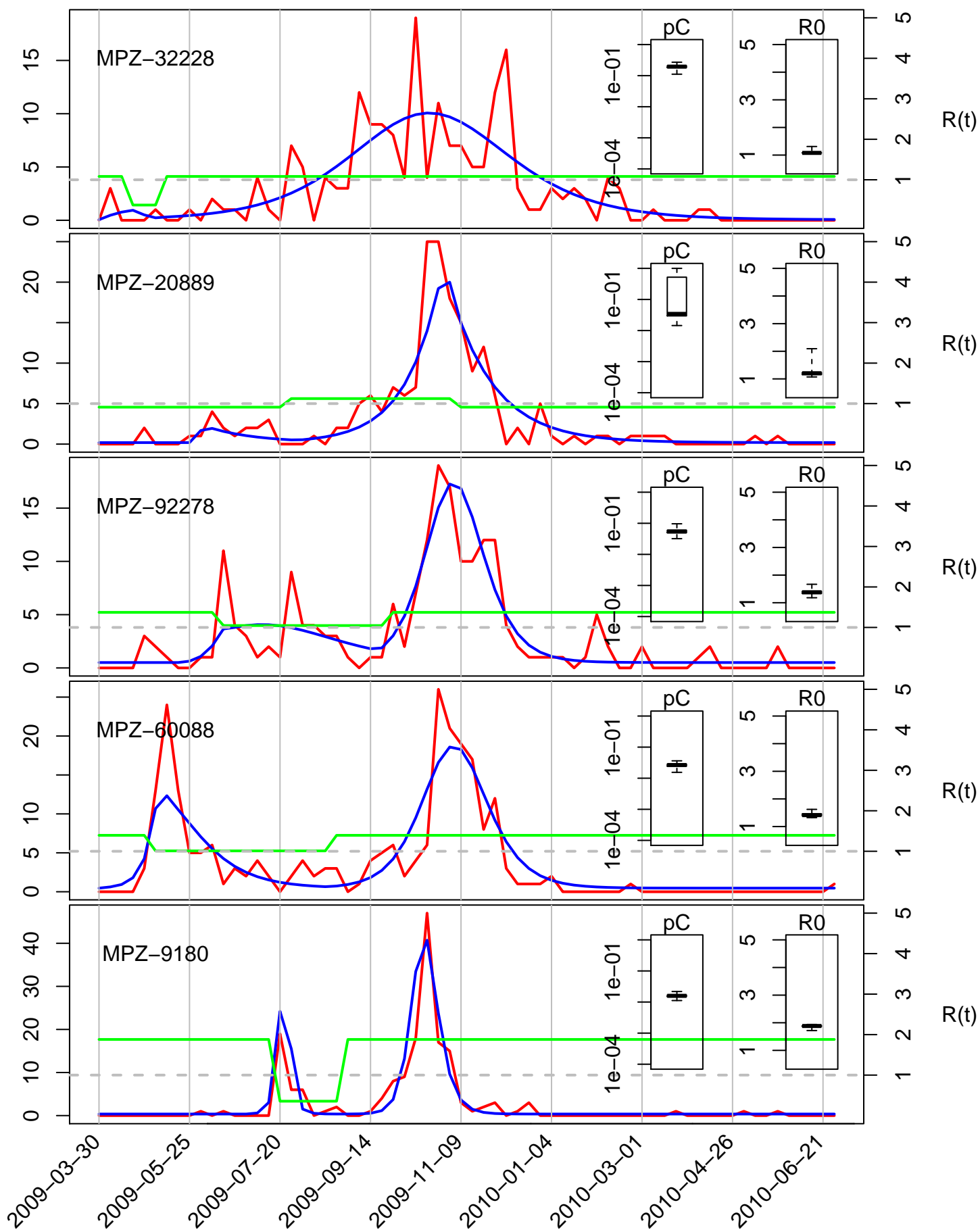

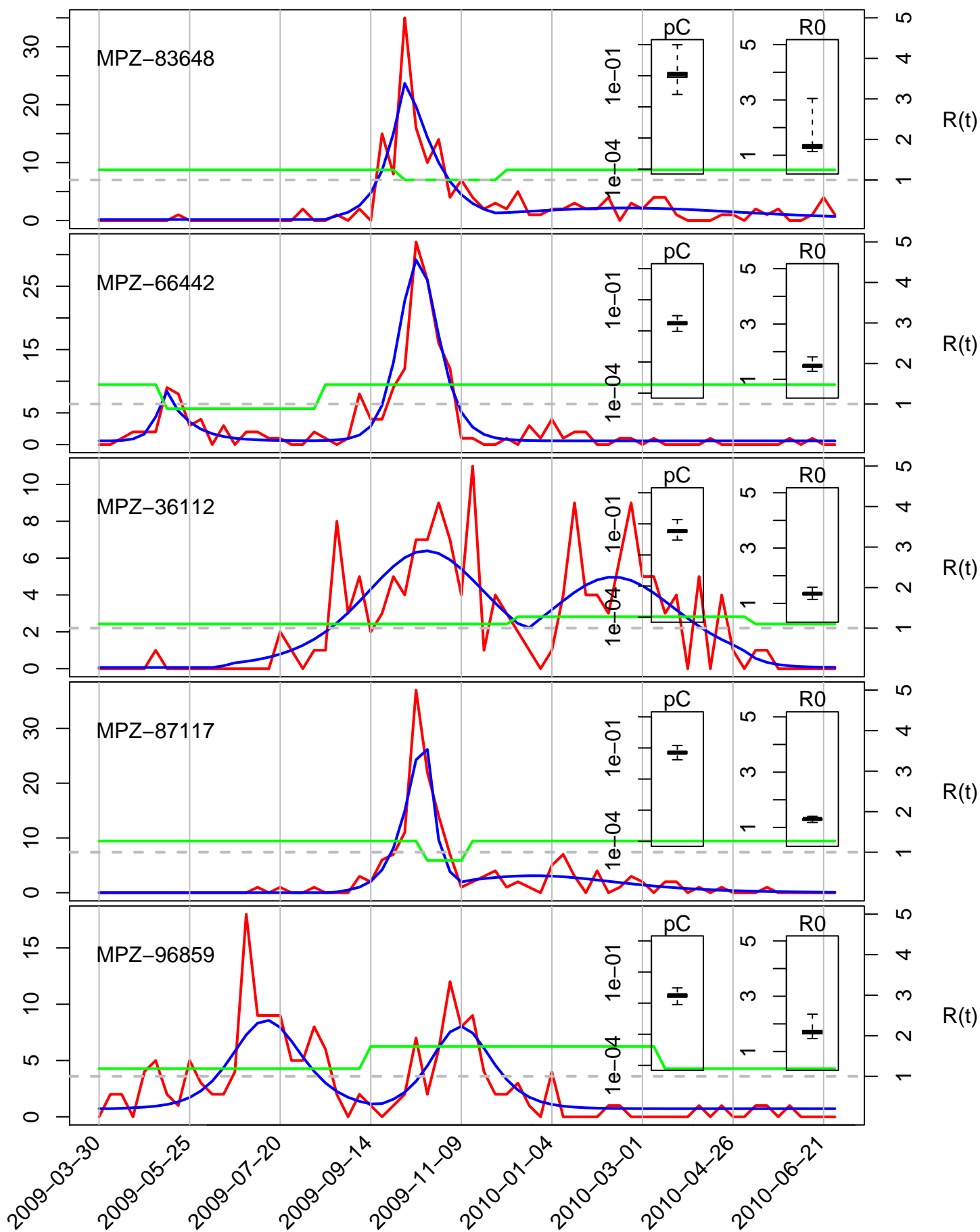

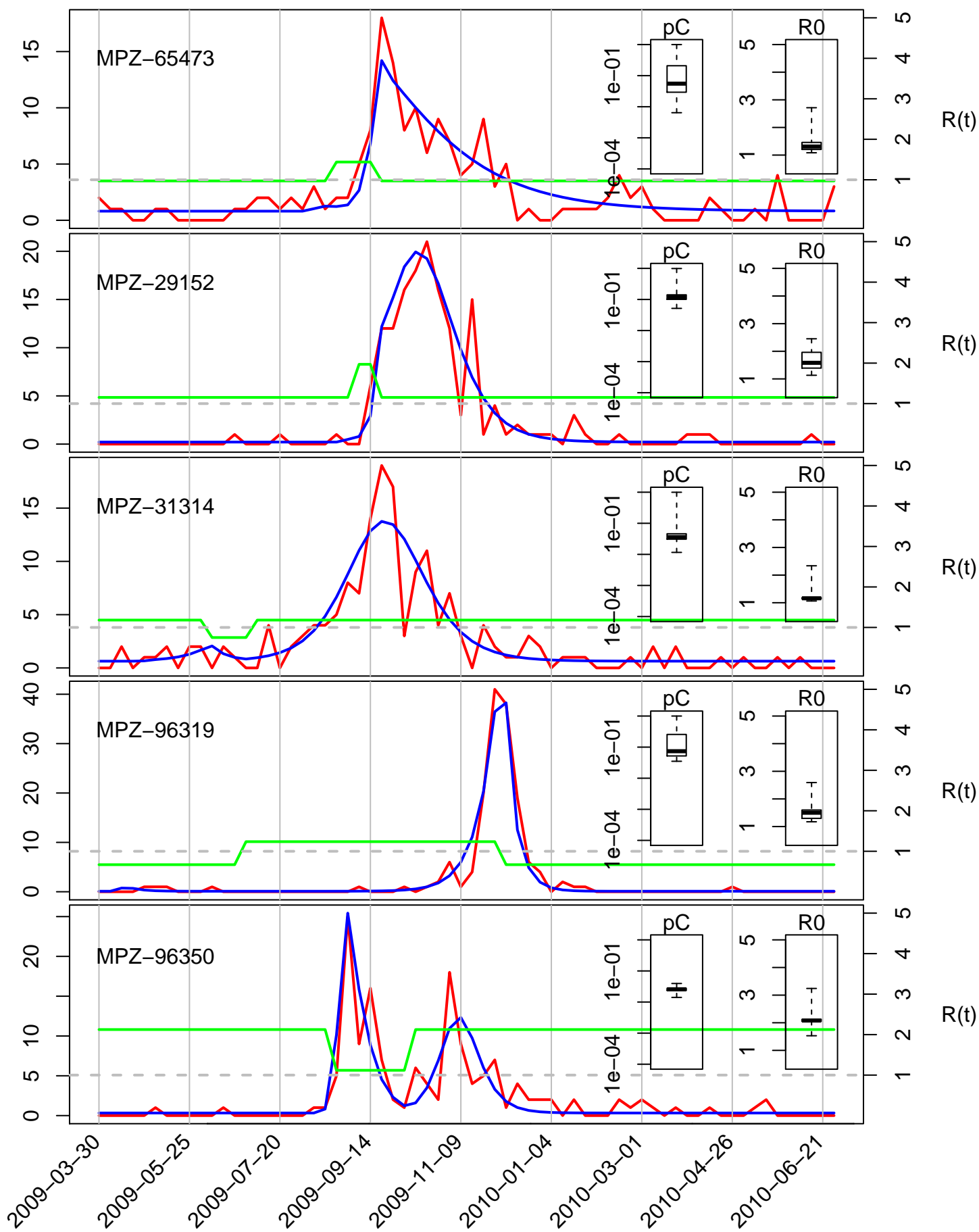

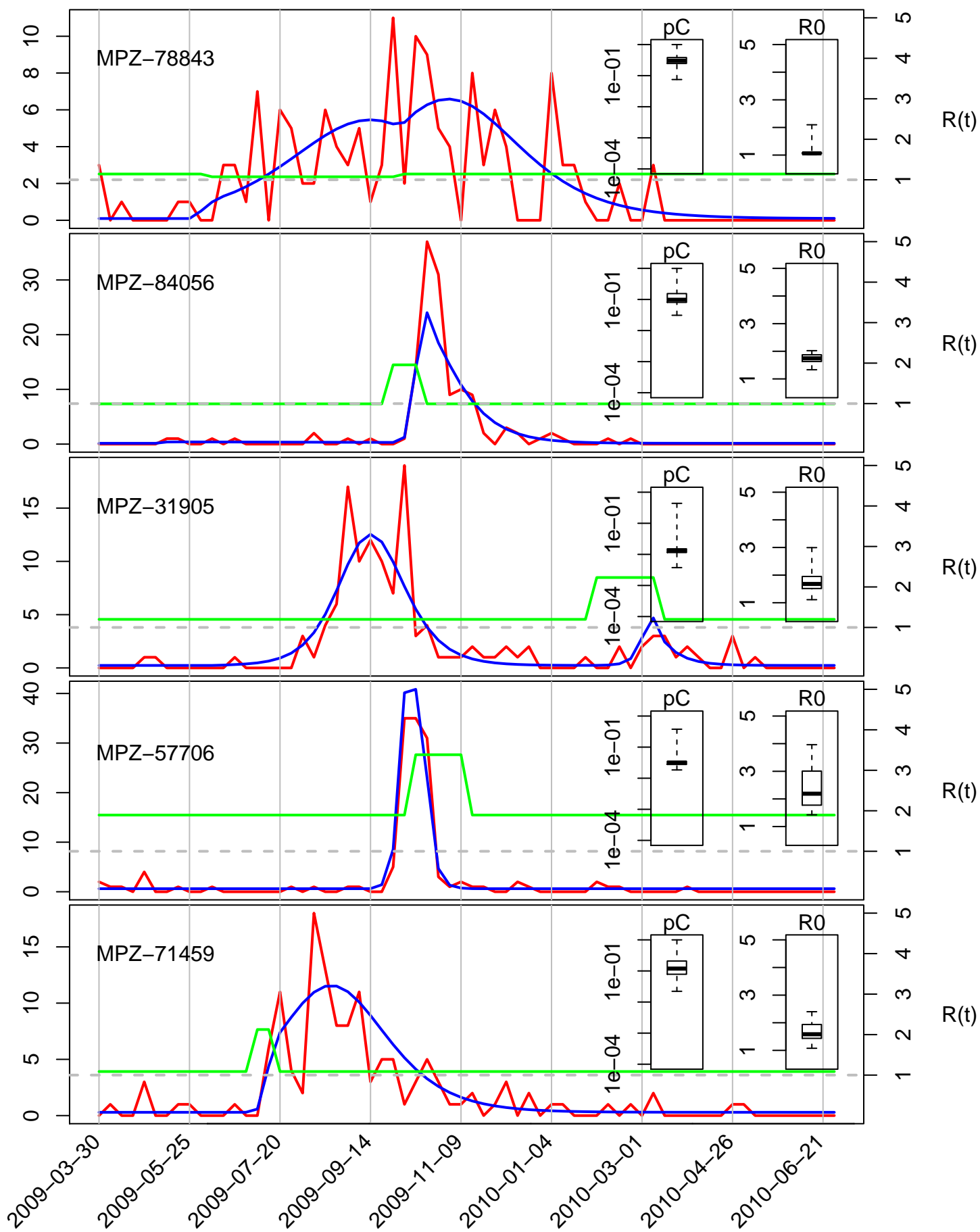

Supplement: S2 Fig — The value of the basic reproduction number is shown in green. A value of 1.0 is indicated by the dashed grey line. The inset shows a box plot of p C and R 0, obtained from the MCMC chain, with the whiskers extending to the extreme values. The military installations are ordered by the total number of ILI cases reported. (PDF) [file pcbi.1004392.s004.pdf]

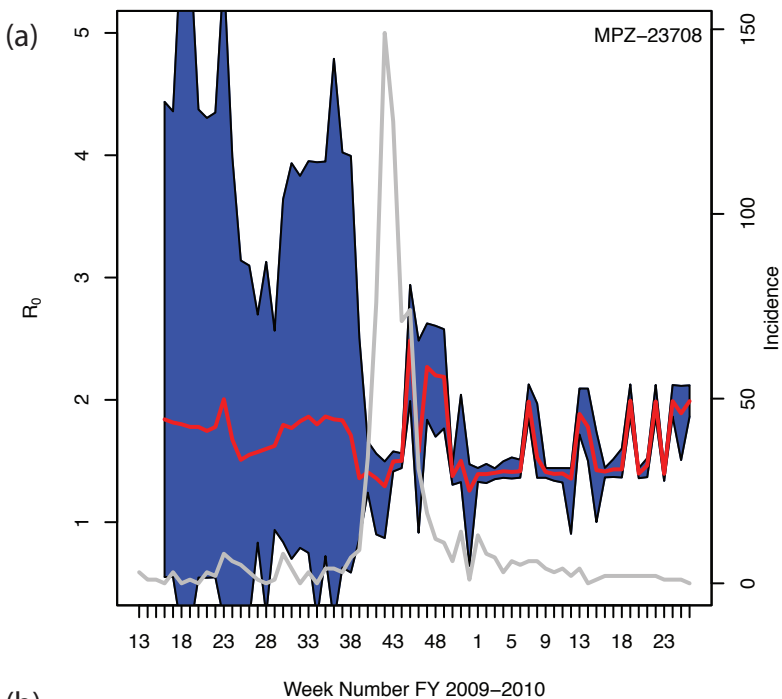

(b)

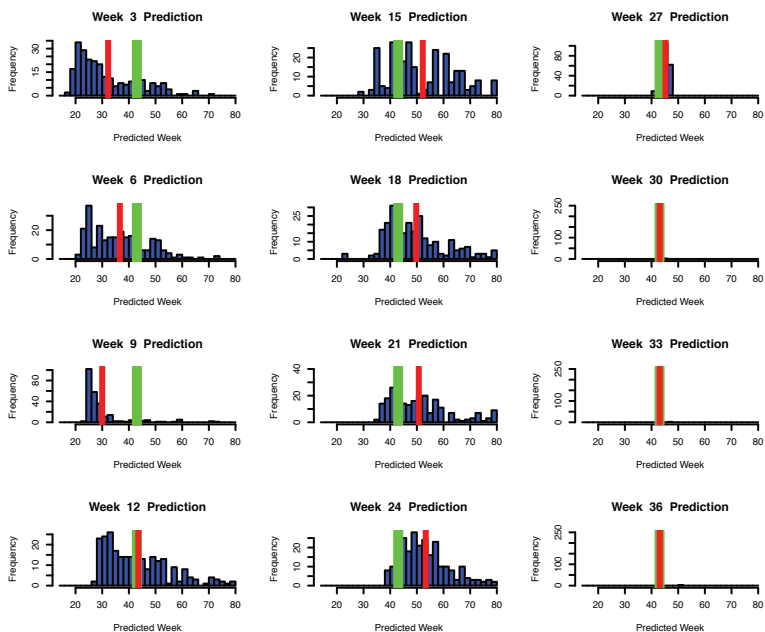

Supplement: S4 Fig — (a) The 95% confidence intervals are shown for military installation 23708 as a function of time by running the fitting procedure using data from the first three weeks, then four weeks, then five weeks, etc., until the full 66 weeks are used. The red curve shows the mean value and the grey line, together with the y-axis on the right-hand-side indicates the ILI profile. Thus, the accuracy substantially improves one week after the exponential rise portion of the outbreak is observed. (b) A second measure of the uncertainty can be estimated from the model’s ability to predict the peak week within ±1 week (e.g., [34]). Here we show results using 250 random selections from the MCMC chains for the same MPZ (23708). The panels show histograms for the MCMC forecast peak timing for predictions made with 3, 6, 9, etc., data points. The actual peak for this installation occurred at week 43 and is marked in green. The red vertical line marks the average of the MCMC ensembles (each of which is shown in blue). Thus, we conclude that only about 3 weeks before the peak occurs do all the predictions collapse down to what will be the observed peak week. This is consistent and complementary to the results shown in (a). (PDF) [file pcbi.1004392.s006.pdf]
