# Supplementary material for: Early Characterization of the Severity and Transmissibility of Pandemic Influenza Using Clinical Episode Data from Multiple Populations
Source: PLoS Comput Biol. 2015 Sep 24;11(9):e1004392. doi: 10.1371/journal.pcbi.1004392 (PMC4581836; doi:10.1371/journal.pcbi.1004392)
Supplement: S2 Table — (PDF) [file pcbi.1004392.s002.pdf]

Table S2: Model fit parameters for the top-50 MPZs.

Rank	MPZ^1	N^2_{total}	t^3_0 (weeks)	t^4_1 (weeks)	Δt^5 (weeks)	Baseline[6]	p^7_C	R^{A8}_0	R^{B9}_0	AIC^{10}_c
1	23708	15842	23.7 [22.9, 24.4]	6.55 [5.34, 7.8]	6.26 [5.25, 7.52]	2.15 [1.76, 2.61]	0.121 [0.105, 0.139]	1.4 [1.35, 1.48]	1.12 [1.08, 1.17]	330 [326, 338]
2	80913	24116	14.7 [12.5, 16.3]	13.5 [12, 15.4]	1.92 [1.08, 12.2]	2.98 [2.58, 3.42]	0.0588 [0.0413, 0.0673]	1.18 [1.15, 1.21]	1.47 [1.36, 1.72]	379 [375, 387]
3	78236	24823	1.18 [1.02, 1.71]	23.3 [22.3, 23.9]	1.67 [1.05, 3.03]	1.43 [1.06, 1.86]	0.117 [0.104, 0.129]	1.07 [1.07, 1.08]	1.37 [1.26, 1.68]	393 [388, 400]
4	92134	14775	1.1 [1.01, 1.48]	25.5 [24.7, 25.9]	22.2 [13.5, 37.6]	1.88 [1.46, 2.36]	0.0598 [0.0518, 0.0687]	1.13 [1.12, 1.14]	1.53 [1.46, 1.61]	392 [388, 400]
5	23665	11554	23 [22.3, 23.7]	11.5 [10.5, 12.7]	18.2 [8.35, 37.1]	1.96 [1.64, 2.34]	0.0574 [0.0504, 0.0655]	1.4 [1.35, 1.45]	2.07 [1.86, 2.32]	312 [308, 319]
6	92055	26561	2.26 [1.4, 4.44]	13.2 [12.1, 13.8]	1.25 [1.02, 1.74]	1.13 [0.748, 1.62]	0.0806 [0.0691, 0.09]	1.14 [1.13, 1.15]	0.0209 [0.00159, 0.0862]	404 [399, 411]
7	99506	9781	11.9 [6.77, 13.7]	9.19 [2.54, 31.1]	1.5 [1.03, 12.2]	0.888 [0.567, 1.26]	0.244 [0.17, 0.55]	1.09 [0.988, 1.14]	1.48 [1.01, 1.82]	286 [280, 298]
8	29207	24012	1.36 [1.14, 5.72]	9.27 [4.26, 9.93]	3.1 [2.32, 4.37]	0.956 [0.605, 1.5]	0.887 [0.412, 0.992]	0.959 [0.951, 0.97]	1.5 [1.34, 1.54]	405 [399, 430]
9	78234	15355	8.88 [8.63, 9.09]	4.48 [4.05, 4.95]	9.01 [8.28, 9.71]	1.29 [1.01, 1.61]	0.0518 [0.0468, 0.0574]	1.5 [1.49, 1.52]	1.12 [1.11, 1.13]	360 [357, 368]
10	32542	10337	14.1 [13, 15.6]	12.3 [10.5, 13.9]	2.64 [1.16, 8.98]	0.827 [0.455, 1.19]	0.149 [0.122, 0.2]	1.18 [1.15, 1.21]	0.981 [0.726, 1.06]	272 [267, 280]
11	42223	15019	13.3 [1.18, 20.3]	11.1 [1.85, 21.7]	4.21 [1.18, 10.7]	0.43 [0.0805, 0.776]	0.15 [0.094, 0.391]	1.05 [0.973, 1.13]	1.31 [1.17, 1.86]	227 [222, 235]
12	23511	7254	23.2 [22.6, 23.8]	17.7 [15.6, 20.4]	6.85 [3.88, 9.94]	0.398 [0.244, 0.6]	0.0963 [0.0851, 0.108]	1.33 [1.3, 1.37]	2.16 [1.91, 2.61]	236 [232, 244]
13	76544	32831	1.93 [1.07, 5.28]	23.6 [19.2, 29.7]	1.49 [1.03, 20.2]	0.816 [0.191, 1.18]	0.0457 [0.0379, 0.161]	1.09 [1.08, 1.11]	1.34 [0.961, 1.51]	285 [280, 294]
14	79920	20999	15.9 [15.3, 16.8]	5.25 [4.12, 5.94]	2.9 [1.49, 5.71]	1.82 [1.51, 2.17]	0.0228 [0.0189, 0.0263]	1.41 [1.37, 1.51]	0.442 [0.0175, 0.883]	298 [294, 306]
15	22134	11588	7.15 [7.06, 7.23]	1.43 [1.04, 1.93]	6.04 [5.34, 6.72]	1.2 [0.973, 1.46]	0.0246 [0.0219, 0.0274]	2.65 [2.5, 2.78]	1.08 [1.04, 1.12]	378 [374, 385]
16	89191	12110	22.5 [18, 24.4]	9.73 [1.44, 13.3]	5.63 [1.07, 31.7]	0.626 [0.43, 0.85]	0.053 [0.0357, 0.135]	1.32 [0.951, 1.7]	1.31 [0.687, 2.01]	213 [206, 224]
17	28547	10719	1.99 [1.07, 4.1]	11.4 [7.82, 13.5]	3.69 [1.1, 10.8]	1.23 [0.745, 1.78]	0.109 [0.0714, 0.151]	1.12 [1.09, 1.17]	0.878 [0.164, 1.02]	305 [301, 312]
18	94535	13454	2.3 [1.11, 5.75]	12.6 [8.61, 15]	1.99 [1.06, 8.06]	1.7 [1.13, 2.34]	0.071 [0.0386, 0.127]	1.12 [1.07, 1.19]	0.727 [0.129, 1.02]	301 [297, 310]
19	98431	20071	5.03 [4.04, 6.02]	5.26 [3.73, 6.39]	12 [9.63, 14.9]	1.09 [0.788, 1.45]	0.022 [0.0166, 0.0276]	1.42 [1.33, 1.63]	0.945 [0.843, 1.03]	279 [274, 292]
20	28310	28763	4.03 [2.88, 4.62]	9.82 [5.8, 13.6]	16.2 [10.9, 21.1]	0.0109 [0.00017, 0.518]	0.869 [0.499, 0.99]	0.965 [0.954, 0.973]	1.07 [1.05, 1.11]	263 [258, 272]
21	32214	12910	6.49 [1.12, 12.7]	3.7 [1.12, 34.5]	2.33 [1.07, 30.2]	0.897 [0.546, 1.27]	0.0811 [0.0603, 0.212]	1.11 [0.98, 1.13]	0.974 [0.198, 1.36]	264 [261, 271]
22	85309	7996	25.2 [24.3, 25.7]	4.41 [3.21, 5.7]	5.42 [3.69, 6.91]	0.215 [0.0996, 0.402]	0.0646 [0.0536, 0.0774]	1.59 [1.47, 1.76]	1.14 [1.04, 1.24]	170 [165, 178]
23	30905	20883	1.55 [1.04, 2.85]	25.5 [1.31, 36.8]	12.5 [1.16, 33.6]	0.273 [0.00212, 0.536]	0.0682 [0.0492, 0.554]	1.09 [0.939, 1.14]	0.974 [0.129, 1.18]	240 [235, 249]
24	20762	14416	6.15 [1.4, 16.3]	11.7 [4.71, 25.6]	12.1 [2.86, 17.2]	0.315 [0.0017, 0.699]	0.413 [0.0791, 0.949]	0.935 [0.893, 1.05]	1.16 [1.1, 1.32]	219 [212, 228]
25	22060	14138	3.14 [1.12, 9.1]	25.1 [18.6, 27.8]	2.37 [1.34, 4.25]	0.656 [0.432, 0.948]	0.0421 [0.0333, 0.0527]	1.06 [1.04, 1.08]	1.62 [1.41, 1.75]	219 [215, 228]
26	40121	14289	14.9 [2.09, 16.3]	20.4 [1.26, 24.8]	17.5 [3.39, 34.8]	0.673 [0.466, 0.923]	0.451 [0.0779, 0.934]	1.05 [0.603, 1.1]	0.549 [0.0969, 1.11]	282 [275, 291]
27	39534	12782	5.12 [1.38, 17.9]	9.43 [2.99, 17.1]	16.6 [1.23, 20.6]	0.665 [0.257, 1.18]	0.571 [0.0347, 0.959]	0.942 [0.889, 1.23]	1.09 [0.437, 1.12]	283 [275, 295]
28	23801	7683	8.33 [2.58, 15.2]	5.11 [1.56, 14.1]	2.11 [1.13, 12.6]	0.402 [0.238, 0.627]	0.0805 [0.0658, 0.108]	1.18 [1.07, 1.21]	0.546 [0.0804, 1.33]	222 [216, 232]
29	29905	4090	9.33 [8.29, 10.6]	5.7 [4.16, 7.85]	7.11 [1.54, 10.1]	0.15 [0.0634, 0.294]	0.146 [0.115, 0.178]	1.25 [1.2, 1.35]	0.95 [0.439, 1.03]	276 [272, 284]
30	23604	11994	24.3 [23.2, 24.8]	10.9 [10.1, 12.1]	15.7 [6.25, 36.3]	0.284 [0.168, 0.437]	0.021 [0.0177, 0.0257]	1.44 [1.36, 1.48]	2.43 [1.99, 2.83]	201 [198, 209]
31	32228	6860	1.15 [1.01, 1.52]	1.55 [1.04, 4.02]	1.56 [1.04, 5.25]	0.003 [0.000136, 0.117]	0.195 [0.163, 0.228]	1.08 [1.07, 1.09]	0.615 [0.306, 0.963]	245 [242, 252]
32	20889	16676	7.27 [2.37, 10.5]	7.16 [4.36, 25.4]	7.76 [1.36, 15.2]	0.347 [0.162, 0.608]	0.0343 [0.0251, 0.944]	1.2 [0.876, 1.25]	0.947 [0.255, 1.68]	208 [200, 218]
33	92278	6709	8.07 [1.76, 8.9]	3.47 [1.33, 4.79]	14 [11.6, 22.8]	0.566 [0.339, 0.823]	0.0544 [0.0438, 0.0687]	1.38 [1.29, 1.47]	1.05 [0.999, 1.09]	245 [240, 254]
34	60088	19921	1.4 [1.06, 2.19]	5.27 [4.15, 5.92]	14.7 [13.1, 17]	0.184 [0.0242, 0.522]	0.0265 [0.0212, 0.0312]	1.41 [1.38, 1.53]	1.02 [0.97, 1.06]	241 [237, 248]
35	9180	13848	14.2 [14, 14.3]	3.49 [3.04, 3.94]	5 [4.31, 5.68]	0.335 [0.204, 0.504]	0.0158 [0.0137, 0.0183]	1.88 [1.83, 1.92]	0.344 [0.272, 0.436]	192 [189, 199]
36	83648	5215	21.6 [20.5, 22.7]	6.69 [5.06, 8]	7.8 [6.07, 9.98]	0.227 [0.0723, 0.485]	0.116 [0.0834, 0.15]	1.29 [1.23, 1.39]	1.03 [0.984, 1.09]	221 [216, 229]
37	66442	15552	2.86 [1.93, 3.49]	4.6 [4.04, 5.76]	11.7 [8.14, 14.2]	0.721 [0.492, 0.983]	0.0177 [0.0142, 0.0223]	1.49 [1.38, 1.57]	0.838 [0.594, 0.958]	231 [226, 238]
38	36112	8100	15.1 [13.1, 16.4]	1.54 [1.05, 2.89]	22.8 [20.2, 24.8]	0.0383 [0.0026, 0.171]	0.0591 [0.0481, 0.0748]	1.34 [1.26, 1.41]	1.11 [1.09, 1.13]	213 [209, 221]
39	87117	7567	22 [20.7, 23]	7.78 [6.4, 9.63]	3.97 [2.45, 5.41]	0.105 [0.0319, 0.234]	0.0708 [0.0577, 0.0877]	1.31 [1.24, 1.37]	0.909 [0.764, 0.994]	179 [174, 187]
40	96859	15517	1.58 [1.04, 3.4]	23.8 [22, 25.5]	17.8 [7.11, 36.9]	0.599 [0.358, 0.888]	0.0176 [0.0133, 0.0225]	1.18 [1.16, 1.22]	1.7 [1.59, 1.89]	242 [238, 249]
41	65473	15807	13.6 [2.92, 20.3]	8.4 [1.4, 31.4]	3.78 [1.27, 15.2]	0.839 [0.547, 1.14]	0.0587 [0.0168, 0.725]	1.02 [0.931, 1.24]	1.33 [0.734, 1.91]	228 [222, 237]
42	29152	4531	21.6 [18.8, 23.3]	2.33 [1.08, 19.2]	1.86 [1.06, 35.3]	0.182 [0.0805, 0.322]	0.119 [0.0814, 0.191]	1.15 [1.04, 1.56]	1.54 [1.09, 2.12]	161 [156, 171]

43	31314	13860	4.95 [1.24, 13.6]	6.14 [1.29, 33]	4.25 [1.14, 29.2]	0.588 [0.333, 0.864]	0.0368 [0.0252, 0.497]	1.15 [0.918, 1.2]	1.03 [0.174, 1.36]	216 [210, 223]
44	96319	4658	28.3 [21.8, 29.7]	3.36 [1.59, 11.9]	2.53 [1.1, 29.4]	0.152 [0.0717, 0.268]	0.0516 [0.0421, 0.134]	1.46 [0.866, 1.69]	1.21 [0.288, 1.71]	125 [120, 134]
45	96350	7185	20.3 [18.4, 20.6]	2.31 [1.16, 2.93]	6.24 [5.35, 9.64]	0.437 [0.283, 0.641]	0.0257 [0.0204, 0.0309]	2.08 [1.96, 2.66]	1.19 [1.13, 1.35]	203 [198, 222]
46	78843	3708	7.93 [4.99, 11.1]	23.6 [1.33, 38.6]	4.89 [1.12, 35.2]	0.261 [0.0995, 0.489]	0.302 [0.181, 0.787]	1.06 [1.01, 1.11]	0.924 [0.133, 1.18]	247 [242, 254]
47	84056	7961	5.34 [1.3, 24.8]	21.5 [2.13, 26.3]	2.86 [1.44, 6.45]	0.116 [0.000541, 0.282]	0.0979 [0.0634, 0.511]	0.99 [0.845, 1.02]	1.75 [1.5, 1.97]	138 [134, 145]
48	31905	22656	12.4 [9.83, 15.8]	27 [2.02, 34.6]	9.94 [1.69, 27.9]	0.281 [0.0744, 0.645]	0.0133 [0.00788, 0.0183]	1.21 [1.16, 1.52]	1.7 [1.02, 2.35]	192 [186, 206]
49	57706	4670	7.28 [1.49, 26.3]	2.38 [1.1, 20.5]	10.3 [1.22, 24.8]	0.374 [0.245, 0.537]	0.0331 [0.0244, 0.0407]	1.78 [1.58, 3.58]	0.668 [0.0643, 3.34]	158 [150, 165]
50	71459	8193	8.53 [1.12, 13.6]	6.29 [1.18, 14.4]	1.68 [1.05, 7.83]	0.28 [0.008, 0.521]	0.119 [0.0511, 0.426]	1.04 [0.978, 1.14]	1.58 [1.15, 2.1]	199 [194, 208]
